# Characterization of siderophore producing arsenic-resistant *Staphylococcus sp.* strain TA6 isolated from contaminated groundwater of Jorhat, Assam and its possible role in arsenic geocycle

**DOI:** 10.1186/s12866-018-1240-6

**Published:** 2018-09-04

**Authors:** Saurav Das, Madhumita Barooah

**Affiliations:** 1 Department of Agricultural Biotechnology, Assam Agricultural University, Jorhat, Assam 785013 India; 2 Present Address: Panhandle Research and Extension Centre, University of Nebraska-Lincoln, Scottsbluff, Nebraska 69361 USA

**Keywords:** Arsenic, Arsenate reductase, Siderophore, *Staphylococcus sp*., Jorhat, Assam

## Abstract

**Background:**

Microorganisms specifically bacteria play a crucial role in arsenic mobilization and its distribution in aquatic systems. Although bacteria are well known for their active participation in the different biogeochemical cycles, the role of these bacteria in regulating the concentration of arsenic in Brahmaputra valley has not been investigated in detail.

**Results:**

In this paper, we report the isolation of an arsenic resistant bacterium TA6 which can efficiently reduce arsenate. The isolate identified as S*taphylococcus sp.* TA6 based on the molecular and chemotaxonomic identification (FAME) showed resistance to the high concentration of both arsenate and arsenite (As(III) = 30 mM; As(V) = 250 mM), along with cross-tolerance to other heavy metals viz.*,* Hg^2+^, Cd^2+^, Co^2+^, Ni^2+^_,_ Cr^2+^. The bacterium also had a high siderophore activity (78.7 ± 0.004 μmol) that positively correlated with its ability to resist arsenic. The isolate, *Staphylococcus sp*. TA6 displayed high bio-transformation ability and reduced 2 mM As(V) initially added into As(III) in a period of 72 h with 88.2% efficiency. The characterization of arsenate reductase enzyme with NADPH coupled assay showed the highest activity at pH 5.5 and temperature of 50 °C.

**Conclusions:**

This study demonstrates the role of an isolate, *Staphylococcus sp.* TA6, in the biotransformation of arsenate to arsenite. The presence of *ars* operon along with the high activity of the arsenate reductase and siderophore production in this isolate may have played an important role in mobilizing arsenate to arsenite and thus increasing the toxicity of arsenic in the aquatic systems of the Brahmaputra valley.

**Electronic supplementary material:**

The online version of this article (10.1186/s12866-018-1240-6) contains supplementary material, which is available to authorized users.

## Background

Increasing groundwater arsenic contamination is a concern in many developing countries including Bangladesh and India for its negative health impact [1]. In India, the Brahmaputra river basin is reported to harbor high concentration of geogenic arsenic (As) [2]. Out of the 32 districts in Assam, 23 have been reported to be affected by high arsenic concentration [3, 4]. Titabor subdivision of Jorhat district is considered as one of the most severely arsenic affected areas of Assam with reported As concentration ranging from 194 to 657 μg/l, far above the permissible standard of Bureau of Indian Standards (BIS) (50 μg/l) and World Health Organization (WHO) (10 μg/l) [2, 3, 5] (Additional file 1: Table S1). The very high concentration of arsenic from the Titabor region was also found during the present study (Additional file 1: Table S2). Arsenic is a metalloid widely distributed in the earth’s crust and its concentration can exist from traces to up to hundreds of mg/kg or mg/l in both soil and in water (soil: 01–40 mg/kg; water: 10–5000 μg/l) [6]. In groundwater, the element is predominantly found in two states viz.*,* arsenate (As(V)) and arsenite (As(III)). Arsenate is predominant in the oxic environment and gets strongly absorbed by chemicals like ferric-oxyhydroxide, ferrihydrite, apatite, and alumina. The arsenite form is predominant in the anoxic environment and is more mobile and toxic than arsenate [7]. The geochemical cycling of arsenic is composite in nature; involving several physical and chemical factors along with the biological agents. Bacteria play a critical role in mobilization and speciation of arsenical compounds in aquatic systems [8]. Arsenic resistant bacteria have evolved necessary genetic makeup which confers them with the ability to resist high concentration of arsenic as well as other toxic metalloids [9]. Several strains *Acidothiobacillus, Aeromonas, Bacillus, Deinococcus, Desulfitobacterium, Exiguobacterium, Flavobacterium, Rhodobacter, Arthrobacter, Acinetobacter,* and *Pseudomonas* are reported to tolerate high concentrations of arsenical elements like arsenate and arsenite [10, 11]*.* The involvement of the genus Staphylococcus viz., *Staphylococcus aureus* [12]*, Staphylococcus succinus* [13]*,* and *Staphylococcus sp.* strain NBRIEAG-8 with high arsenic resistance have been previously reported [14]. However, the arbitrated mechanism of arsenic mobilization by bacteria is still poorly understood and needs further investigation to decipher their role in sediment-bound arsenic mobilization. Bacteria can either reduce, oxidize or can methylate the arsenical compounds in a way of resistance or use them in the cellular respiratory pathway [15]. Arsenate reducing bacteria are able to reduce arsenate [As(V)] to arsenite [As (III)] and use the reduced form as an electron acceptor in a respiratory pathway or efflux the same as a mean of resistance mechanisms [16]. Arsenic resistant bacteria are frequently detected with siderophore activity. Siderophore are high-affinity iron chelating compounds produced and secreted by few microorganisms to forage the environmental iron from inorganic phase by formation of soluble Fe^3+^ complex, which can be taken up by active transport mechanisms [17]. The Fe sequestering ability of bacteria through siderophore production confers them with an added advantages over the non-siderophore producers in arsenic resistance. The previous study has shown that the rate of arsenic uptake and reduction efficiency of a bacteria significantly varies with varied siderophore concentration [18].

In this paper, we report the isolation and characterization of an isolate of bacteria that displayed resistance to high concentration of both arsenate and arsenite. Based on its morphological, molecular and chemotaxonomic characterization the isolate was identified as *Staphylococcus sp.* TA6. Besides harboring the *ars* operon, the isolate also produced siderophore and displayed high reduction efficiency (88.2%), reducing the initial 2 mM arsenate [As(V)] added to arsenite [As(III)] over a period of 72 h.

## Methods

### Sample collection and isolation

Contaminated groundwater samples were collected from Tanti-Gaon (GPS: 26.58.101, 94.16.391) (Additional file 2: Figure S1), a village in Titabor subdivision of Jorhat district, Assam, India. The concentration of arsenic in the water samples was measured by atomic absorption spectrophotometer (AAS; PerkinElmer; AAnalyst 400 AA Spectrometer) following the standard protocol as described by Behari and Prakash [19]. The collected samples were enriched in LB broth, subjected to serial dilution and cultured in arsenate amended LB agar plates (10 mM Arsenate/1 mM of Arsenite) and incubated at 30 °C for 48 h. Individual colonies were picked up based on the morphological identities and sub-cultured to obtain the pure isolates (Additional file 3:Figure S2).

### Identification and characterization of new isolate

#### Identification based on 16S rRNA and phylogeny

Genomic DNA was extracted from approximately 100 mg of the cell as per standard phenol-chloroform method. The 1500 bp region of the 16S rRNA gene was amplified from the extracted genomic DNA using the universal primer 5’ TACGGYTACCTTGTTACGACTT 3′ (1492R) [20], 5’ AGAGTTTGATCMTGGCTCAG 3′ (27F) [21]. The amplification was carried out in a reaction with a final volume of 25 μl containing 1.5 μl of template DNA, 1 μl (20pM) of the forward primer, 1 μl (20 pM) of the reverse primer, 2.5 μl (2.5 mM of each) dNTP mix, 2.5 μl of 10× PCR buffer, 1 μl (1 U) of Taq DNA polymerase. A negative control (PCR mix without DNA) was included in all PCR experiments. The PCR reaction conditions were set for 94 °C for 3 min, followed by 30 cycles of denaturation at 94 °C for 30 s, annealing at 58 °C for 1 min and extension at 72 °C for 2 min, before a final extension at 72 °C for 7 min. The PCR products were purified using PureLink™ PCR Purification Kit (Thermo Fischer Scientific, India and sequenced using ABI 3500 8-capillary array sequencer (Applied Biosystems, USA). The forward and reverse sequences obtained were assembled using the Codon-Code Aligner software (version: 5.1). Nucleotide sequence identities were determined using the BLAST tool from the National Center for Biotechnology Information (NCBI) and Similarity index value from EzTaxon Server. The partial sequence data for the 16S rRNA genes have been submitted to GeneBank for further references. Phylogenetic relationship inferred with neighbor-joining (NJ) tree [22]. Sequence divergence among the strains was quantified using Jukes-Cantor distance model [23]. A total of 1000 bootstrap replication were calculated for evaluation of the tree topology.

#### FAME analysis

The fatty acid methyl ester (FAME) profile was analyzed using Sherlock-Midi system and compared with few reference strains of *Staphylococcus* genus for taxonomical validation [24].

#### Minimum inhibitory concentration test

The minimum inhibitory concentration (MIC) of arsenate [As (V)] and arsenite [As (III)] was evaluated to determine the resistance capacity of the isolated bacteria. The bacterial isolates were cultured in freshly prepared LB broth at 30 °C for 48 h and then 100 μl of the freshly cultured bacterial suspension (0.5 McFarland Standard = 1.5 × 10^8^ CFU/ml) was inoculated in minimal salt media (MSM) supplemented with different concentration of arsenite (0.5–30 mM) added as sodium meta-arsenite (m-Na-AsO_2_) and arsenate (10–300 mM) added as disodium hydrogen arsenate (Na_2_HAsO_4_.7H_2_O) and incubated for 72 h at 30 °C and 142 rpm. The microbial growth was recorded with a UV-Visible spectrophotometer at 600 nm.

#### Growth of the bacterial isolate in the presence and the absence of arsenite/arsenate

Among all the isolates, TA6 showed the highest MIC and as such, was taken for studying the growth kinetics in presence and absence of arsenite and arsenate. The isolate was cultured in Luria-Bertani broth containing arsenate in a concentration of 1 mM to 30 mM and arsenite from 0.5 mM to 10 mM respectively. The growth of the isolate was monitored through measurement of the optical density (OD) with a spectrophotometer (Thermo-Scientific, India) at 600 nm (OD600) at a specified interval of time (4 h, 8 h, 12 h, 24 h, 48 h, and 72 h).

#### Cross tolerance

The isolate was tested for its cross-tolerance efficiency with other heavy metals like Hg^2+^ added as HgCl_2_, Cd^2+^ added as CdCl_2_, Co^2+^ added as CoCl_2_, Ni^2+^ added as NiCl_2_ and Al^3+^ added as AlCl_3_ in a concentration ranging from 0.5 to 10 mM in MSM broth culture and absorbance (OD600 nm) was recorded after 48 h to evaluate the bacterial growth.

#### Biochemical tests and carbon source utilization

Biochemical tests for starch hydrolysis, catalase, oxidase, casein production, nitrate reduction, urease, malate, citrate, indole, and motility were done according to the standard protocol described by Krieg [25]. Carbon source utilization was tested using BioMerieux 50 CHB/E strips (BioMerieux, USA).

### Biotransformation assay

#### Qualitative and quantitative biotransformation assay

The ability of the bacteria to reduce As (V) or to oxidize As (III) was evaluated using the silver nitrate (AgNO_3_) method as described by Simeonova et al.*,* [26]. Freshly cultured bacterium grown in minimal salt medium with 5 mM glucose was sub-cultured on two different LB agar plates supplemented with 2 mM of Sodium Meta-Arsenite and Sodium Arsenate respectively and incubated for 48 h at 30 °C. The streaked plates were then flooded with 0.1 M Silver Nitrate (AgNO_3_) solution. Formation of light yellow color will indicate the precipitation of silver ortho-arsenite (Ag_3_AsO_3_) and light brown-red color for precipitation of silver-ortho-arsenate (Ag_3_AsO_4_).

Quantitative assay of arsenate reduction was analyzed by culturing the bacteria in arsenic amended LB broth (2 mM of Arsenate). In a time interval of 6, 12, 24, 48, 72 h the bacterial cells were collected by centrifugation and arsenite content of the supernatant was determined by AAS following standard protocols as described by Aggett and Aspell [27].

#### Arsenate reductase enzyme assay

The enzyme assay was done using NADPH coupled assay as described by Gladysheva et al., [28]. Cell-free crude extracts of *Escherichia sp.* SD23 was used as positive control. Effect of pH and temperature on enzyme activity was also measured using this method.

### Siderophore production and quantification

#### Siderophore production

Production of siderophore was studied using Chrome Azurol S (CAS) agar media as described by Schwyn and Neilands [29]. CAS agar was prepared from four solutions which were sterilized separately before mixing. The **solution I**: Blue dye was prepared by mixing 10 ml of 1 mM FeCl_3_.6H_2_O in 10 mM HCl then with 50 ml of an aqueous solution of 2 mM CAS. The resulting dark purple mixture was added slowly with constant stirring to 40 ml of an aqueous solution of 5 mM Hexa-Decyl Tri-Methyl Ammonium [HDTMA]. The dark blue solution was produced which was autoclaved and then cooled to 50 °C. All reagents in the indicator solution were freshly prepared for each batch of CAS agar. **Solution II:** CAS agar was prepared by dissolving 30.24 g of Piperazine-N, N′-bis ethane sulfonic acid (PIPES) in 750 ml of a salt solution containing 0.3 g KH_2_PO_4_, 0.5 g NaCl, and 1.0 g NH_4_Cl. The pH was adjusted to 6.8 with 50% KOH, and water was added to bring the volume to 800 ml and autoclaved after adding 15 g of agar, and then cooled to 50 °C. **Solution III**: Mix Solution containing the followings: 2 g glucose, 2 g mannitol, 493 mg MgSO_4_,7H_2_O, l mg CaCl_2_, 1.17 mg MnSO_4._7H_2_O, 1.4 mg H_3_BO_3_, 0.04 mg CuSO_4_.5H_2_O, 1.2 mg ZnSO_4_.7H_2_O, and 1.0 mg Na_2_MoO_4_.2H_2_O, was autoclaved, cooled to 50 °C then added to the buffer solution along with 30 ml filter-sterilized 10% (*w*/*v*) casamino acid (**Solution IV**). The indicator solution was added last, with sufficient stirring to mix the ingredients without forming bubbles. CAS agar plates were inoculated with bacterial isolate and incubated at 30 °C for 7 days. Colonies showing orange hollow zone following incubation were recognized as siderophore positive [30].

#### Siderophore quantification

The method of Alexander et al. [31] was used to measure siderophore production in vitro. The bacterial cells were grown at 30 °C for 24 h in 50 ml of Chrome Azurol S (CAS) medium with 5 mM MES (2-(N-morpholino-ethane-sulfonic acid) – KOH buffer at pH 6.8. After the culture growth attains exponential phase at OD-600, the cells were pelleted by centrifugation at 10,000 g for 10 min and the supernatant was filtered through 0.25 μm filter. Siderophore concentration in the filtrate was measured by mixing 500 μl of modified CAS assay solution with 500 μl filtrates. The standard solution of deferoxamine-mesylate was used for siderophore quantification. The sterile CAS-MES-KOH solution was used as a reference solution, which did not contain siderophores. A standard curve was prepared by analyzing the absorbance (630 nm) of the reference solution (A/Aref) as a function of the siderophore concentration.

#### Resistance to arsenic in comparison to siderophore mutant

The role of siderophore in arsenic tolerance was determined following the protocol described by Ghosh et al., [18] using one siderophore mutant (non-producer) *Pseudomonas putida* (Lp10L02M) and one control *Acinetobacter guillourie* (S02Ar2) with low siderophore production ability (10.8 μmol). Arsenic tolerance of the isolate was measured as a percentage of growth rate and As(V) reduction at 5 and 10 mM of As(V) modified LB medium incubated at 30 °C for 24 h shaking at 142 rpm and compared with the TA6. Growth was measured as OD at 600 nm on UV–Vis spectrophotometer. All the data were taken in triplicates.

## Results

### Groundwater sample

The contaminated groundwater samples collected from Titabor subdivision had pH 6.2–7.3 and arsenic concentration of 50–356 μg/l.

### Isolation of arsenic-resistant bacteria and MIC

The enriched groundwater sample was inoculated in arsenate amended LB medium and morphologically different bacterial colonies were picked up and tested for minimum inhibitory concentration of arsenate and arsenite. Among the isolates, TA6 showed highest MIC and was able to grow in medium with 250 mM of arsenate and 30 mM of arsenite.

### Chemotaxonomic and molecular identification with phylogeny

**Fig. 1:**
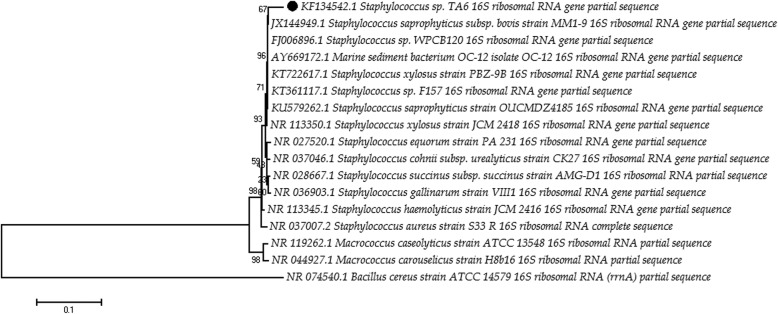
The evolutionary history was inferred using the Neighbor-Joining method. The optimal tree with the sum of branch length = 0.90052 (~ 0.1) is shown. The percentage of replicate trees in which the associated taxa clustered together in the bootstrap test (1000 replicates) are shown next to the branches. The tree is drawn to scale, with branch lengths in the same units as those of the evolutionary distances used to infer the phylogenetic tree. The evolutionary distances were computed using the Jukes-Cantor method and are in the units of the number of base substitutions per site. All positions containing gaps and missing data were eliminated

The 16S rRNA sequence similarity search identified the isolate as one of the species of the genus *Staphylococcus* having 98% pairwise similarity with *Staphylococcus saprophyticus subsp. Bovis* MM19 and *Staphylococcus saprophyticus* strain OUCMDZ4189. Fatty acid methyl ester profile showed most of the fatty acids are branched chains like anteiso C15, anteiso C17, and iso C15. Comparative studies with the fatty acid profile of *S. xylosus, S. cohnii,* and *S. saprophyticus* showed considerable differences of C17:0, iso C17:0, iso C18:0 (Table 1). Therefore, based on both molecular and chemotaxonomic data the bacterium was identified as *Staphylococcus sp.* and the sequence was submitted under the GeneBank accession: KF134542.1 for further references. Phylogenetic analysis showed significant evolutionary difference among the other member of the *Staphylococcus* genus but with the similar lineage of origin (Fig. 1). Evolutionary distance computed with Jack Cantor model and 1000 bootstrap value showed TA6 is 67% homologous on evolutionary lineage with *Staphylococcus saprophyticus subsp. Bovis* MM19.

**Table 1:** Cellular Fatty acid Profile of isolateTA6 (1) and *S. xylosus*(2) *S. cohnii*(3) and *S. saprophyticus*(4)

(% of total fatty acids)				
Straight chain fatty acids	1	2	3	4
C15:0	–	–	–	–
C16:0	1.61	3.44	3.21	3.57
C17:0	0.07	–	–	–
Branched chain fatty acids				
iso C14:0	0.96	2.40	2.48	1.88
iso C15:0	14.04	23.73	15.01	21.09
anteiso- C15:0	50.58	39.24	43.28	41.55
iso C16:0	2.06	0.97	3.14	1.94
iso C17:0	6.80	–	–	–
anteiso - C17:0	13.13	4.48	10.18	4.66
iso C18:0	0.47	–	0.62	–

### Bacterial growth in presence of arsenate and arsenite

**Fig. 2:**
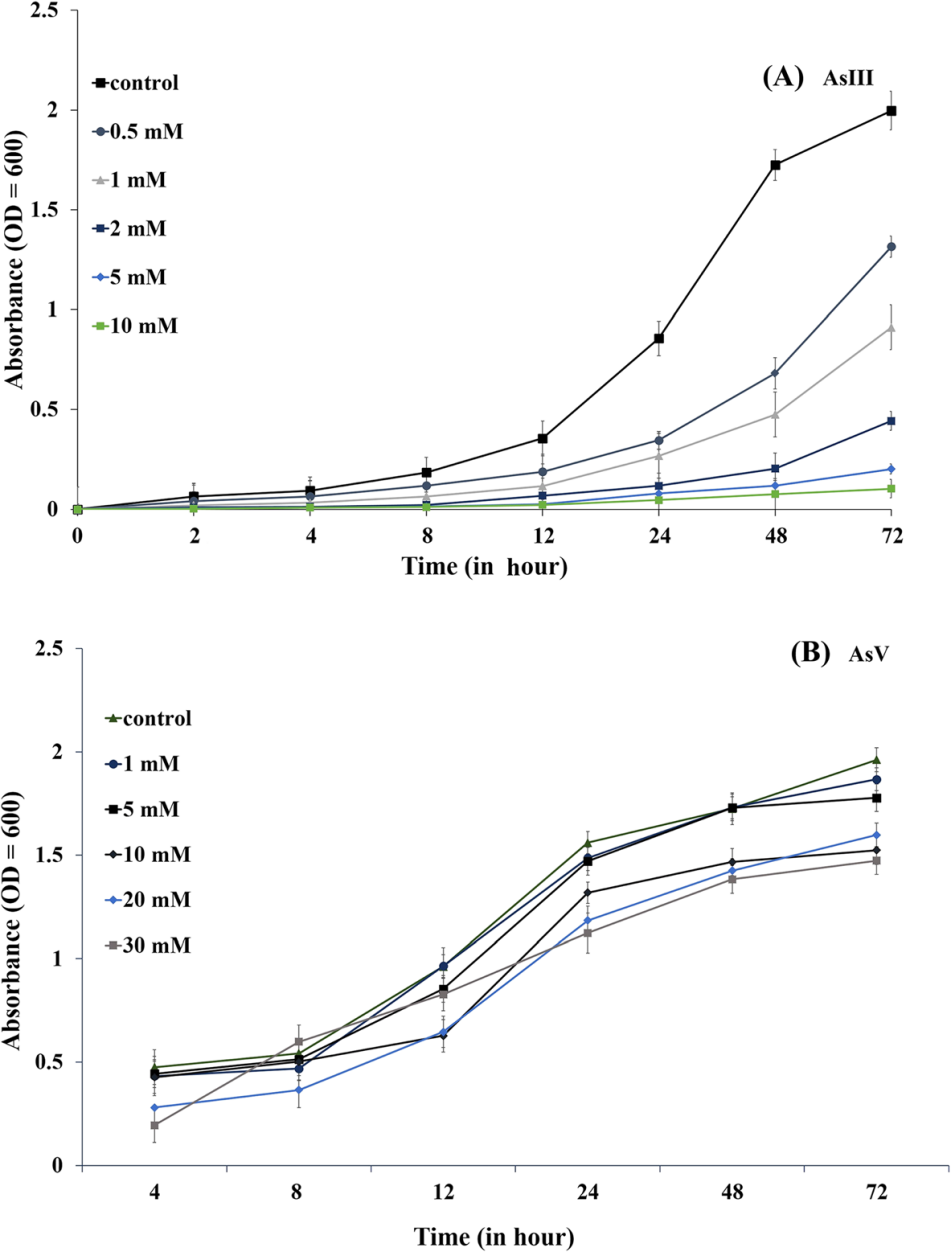
Effect of arsenic on bacterial growth rate (**a**) Arsenite (As(III)) (**b**) Arsenate (As(V))

Growth curve analysis showed the effect of arsenate and arsenite in the bacterial growth pattern. The isolate TA6 was cultured in fresh LB broth with a concentration of arsenate varying from 1 mM – 30 mM and arsenite from 0.5 mM – 10 mM respectively. Bacterial growth was not much affected in the presence of arsenate as compared with control. However, the presence of arsenite in the medium greatly affected the rate of growth. In the presence of arsenate, TA6 started doubling at the lowest time of 4 h but in the presence of arsenite, it took approximately 24 h to start multiplying. At the highest concentration of arsenate (~ 30 mM) taken for the test and at 72 h of incubation time, OD was measured as 1.474 ± 0.067 and for control OD was recorded 1.962 ± 0.058 at the same time of incubation (Fig. 2b). While, at 72 h of incubation in the presence of 10 mM of arsenite growth was reduced when compared to the control. For control, OD was recorded as 1.962 ± 0.058, whereas in the 10 mM of arsenite, the growth was recorded as OD 0.1036 ± 0.043. At lowest concentration of arsenite 0.5 mM, the bacterial cell (TA6) approximately took 8 ± 2 h of incubation to multiply (Fig. 2a).

### Cross tolerance

Other heavy metal tolerance test also showed the resistive capacity of the isolate to various heavy metals like Hg^2+^, Cd^2+^, Co^2+^, Ni^2+^_,_ Cr^2+^. MIC was found as 0.5 mM, 0.8 mM, 1.0 mM, 4 mM, and 6 mM respectively.

### Biochemical test

The bacterium (TA6) was a gram-positive, non-motile, coccus shaped bacterium. It was able to hydrolyze starch, casein and utilize citrate, reduce catalase and showed high siderophore activity (78.7 ± 0.004 μmol) but tested negative for oxidase, nitrate, urease and indole. Carbohydrate utilization test with 50 CHB/E showed it could actively utilize Glycerol, D-Glucose, D-Fructose, Maltose, Lactose, Sucrose, Trehalose, Melezitose, Starch, and D-Turanose.

### Biotransformation assay

**Fig. 3:**
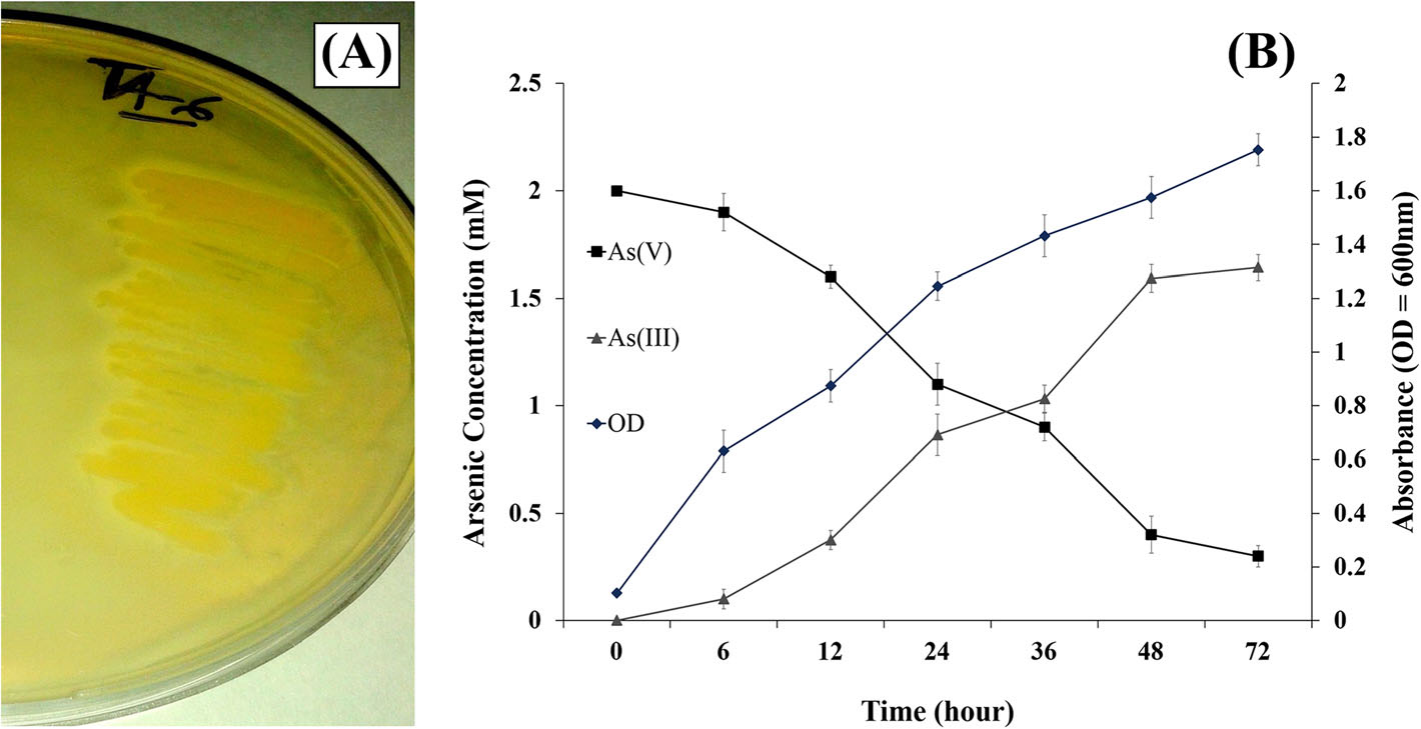
(**a**) Bioconversion of arsenate [As (V)] to arsenite [As (III)]. (**b**) The rate of Biotransformation over an incubation period of 72 h

TA6 was found to be an arsenate reducer. Reduction of arsenate in the petri dish formed a yellow precipitation of silver ortho-arsenite (Ag_3_AsO_3_) which indicates the presence of arsenite (Fig. 3a). In the quantitative assay, it was also found that with a gradual increase in time and with the increased bacterial cell count, the concentration of As(V) gradually decreased with increased concentration of As(III). In a duration of 72 h, nearly 88.2% of the initial 2 mM As(V) is reduced to As(III) (Fig. 3b).

### Arsenate reductase enzyme activity

**Fig. 4:**
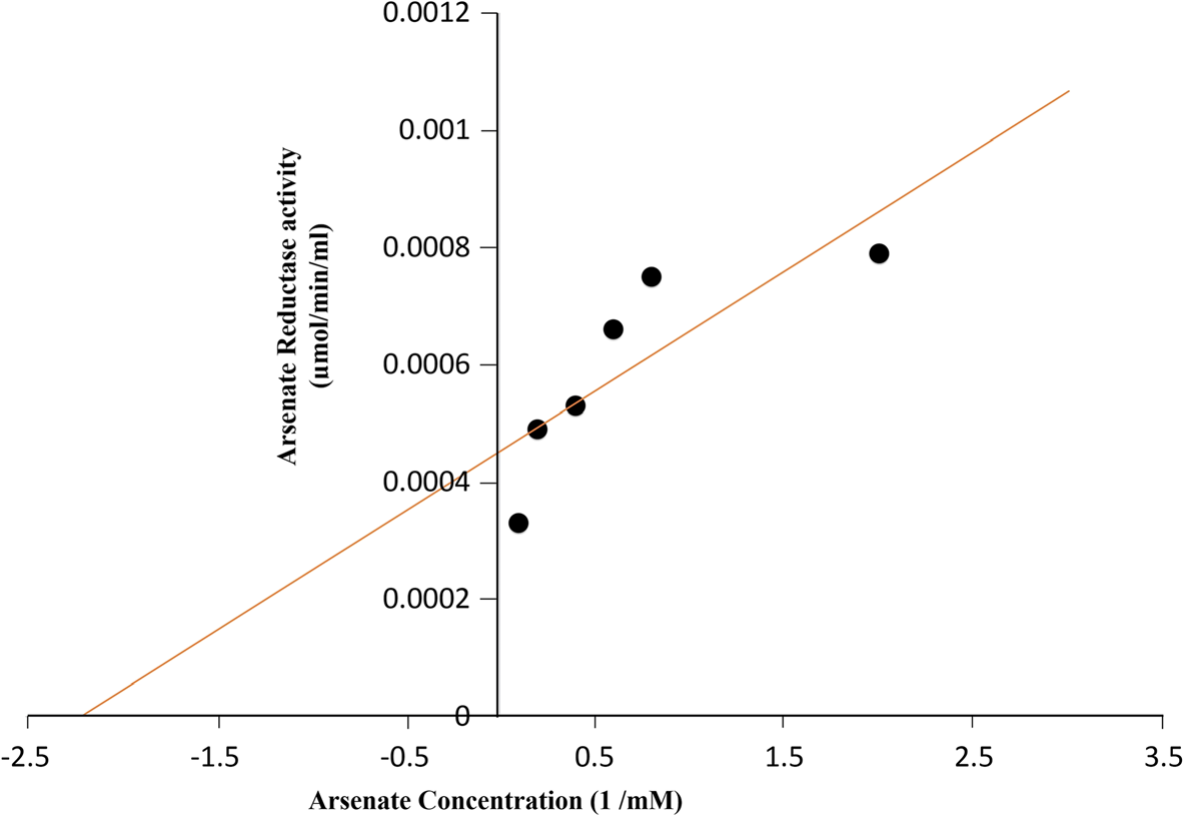
Kinetic Profile of enzyme activity (Lineweaver Burk Plot)

**Fig. 5:**
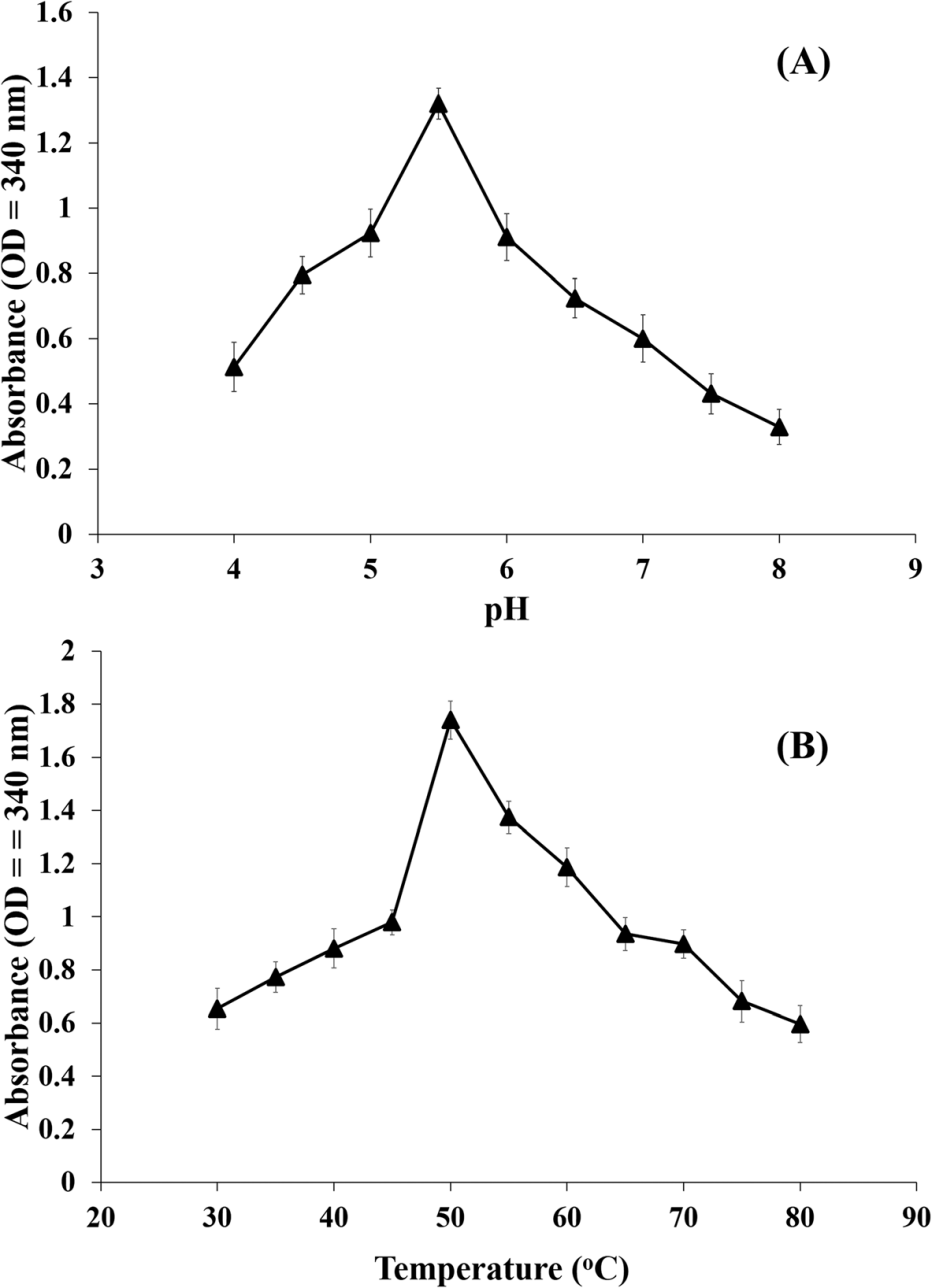
Arsenate Reductase enzyme activity (**a**) At different pH (pH 5.5 was found to be optimal for maximum activity) (**b**) At different Temperature (50 °C was found as the optimal temperature for maximum activity)

Arsenate reductase activity was measured using NADPH coupled oxidation method. A Km of 0.44 mM arsenate and Vmax of 6395 umol/min/ml were measured (Fig. 4). There was no change in activity for 500 μM and 1 mM of arsenate. Temperature and pH are some critical factors for enzyme activity. Temperature-dependent activity assay revealed that 50 °C was the optimal temperature for highest enzymatic activity and in pH-dependent activity assay, pH 5.5 was measured as optimal for highest enzymatic activity (Fig. 5a). Graphical representation of both the data formed a characteristic bell-shaped curved, where initial increased pH and temperature raised the activity till it reaches the optimal point of maximum activity and then the activity was found to gradually cease after the respective optimal value of pH 5.5 and temperature 50 °C (Fig. 5b).

### Siderophore associated arsenate reduction

**Fig. 6:**
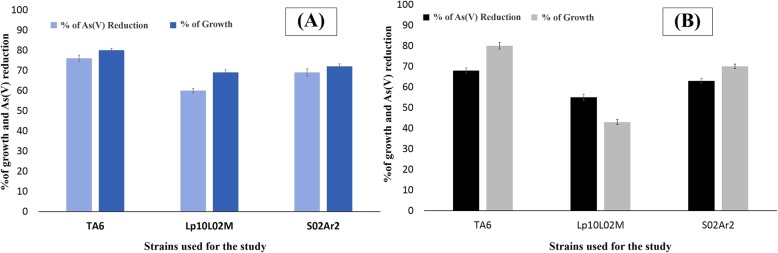
Effect of siderophore on growth percentage and arsenate reduction in the modified LB medium containing (**a**) 5 mM arsenate (**b**) 10 mM arsenate

Microorganisms are the primary chelator of iron which dissociates Fe^3+^ ions with their siderophore activity. Siderophore associated arsenic resistance assay revealed that bacteria with high siderophore TA6 (78.7 ± 0.004 μmol) was significantly a strong As(V) reducer than the mutant strain Lp10L02M (non-producer). The growth of TA6 was also found reflective in comparison to the control and the mutant implying the added resistance ability of the strain to arsenate. In 5 mM and 10 mM arsenate broth, the TA6 showed higher growth as compared to the control strain S02Ar2. However, the mutant strain (Lp10L02M) had slower growth rate as compared to control and showed lesser reduction efficiency (Fig. 6).

## Discussion

Increased arsenic concentration in groundwater has negative impact on the public health due to its carcinogenic nature. The Brahmaputra river basin is considered as one of the severely arsenic contaminated basin in the world [32]. Flood-line areas of the river have been detected with arsenic concentration much above the standard permissible limit set by WHO (10 μg/l) and BIS (50 μg/l) and has become a major health issue for the people residing within these vicinities as they are solely depend on the natural streams and groundwater for potable water. Titabor subdivision of Jorhat district, Assam harbors an alarming concentration of arsenic (194–657 μg/l) [33]. Although, several studies on arsenic poisoning and geogenic distribution of arsenic in this region has been documented [34–36], the role of microbes in the geocycle needs much more attention. Bacteria are known to play important role in the biogeochemical cycle of arsenic and are actively associated with the mobilization of sediment-bound arsenic as indicated from previous studies [37]. Bacteria can interconvert different forms of arsenic through redox reactions and influence the bioavailability, solubility and mobility of arsenical compounds. They employ an array of cellular and metabolic mechanisms including extrusion, entrapment by cellular capsules or by precipitation, oxidation-reduction reaction to resist the toxic concentration of arsenic [8, 38]. Recent evidences indicates to a major role played by bacteria in mobilizing the arsenic in aquatic system [39–41]. As such, it is imperative to investigate and identify the bacteria controlling the biogeochemical cycling of arsenic to design effective strategies to manage arsenic-contamination in aquatic systems.

We isolated a bacterium TA6 from the groundwater sample containing 356 μg/l of arsenic. The isolate was identified as *Staphylococcus sp.* based on the 16S rRNA sequence analysis and fatty acid methyl ester (FAME) profile. Both the 16S rRNA and FAME analysis showed significant differences with the reference strains of *Staphylococcus*. Identity search with the NCBI nr/nt database and EzTaxon server showed an average of 98% identity with the different species of *Staphylococcus* genus. Straight chain fatty acids like C16:0, C17:0 and branched chain fatty acids like iso C14:0, iso C17:0, anteiso C15:0 showed differences when compared with the FAME profile of *S. xylosus, S. cohnii* and *S. saprophyticus* (Table 1). The bacterium survived in arsenate (MIC = 250 mM) and arsenite (MIC = 30 mM). Resistance to arsenite concentration greater than 10 mM and arsenate greater than 100 mM is considered as significantly high, whereas resistance to 200 mM As(V) and 30 mM As(III) is regarded as a hyper-tolerance property [42]. The higher tolerance to inorganic arsenic may be due to the presence of arsenic resistance operonic genes (*ars*R*, ars*B, and *ars*C) as confirmed by PCR detection method (data not shown). The presence of arsenic resistance genes among the members of *Staphylococcus* genus is well documented [43–45]. The bacterium also showed resistance to other heavy metals viz., Hg^2+,^ Cd^2+^, Co^2+^, Ni^2+^_,_ Cr^2+,^ and MIC ranged from ≥0.5–10.0 mM.

Bacteria are known for their ability to produce different biogenic chelating agents like siderophore in the iron-limiting environment. Siderophore solubilizes the ferric iron in the iron-starved environment and transports the Fe^3+^ into the cell [46]. Siderophore enables the bacteria to grow in an environment where iron is the limiting factor [47]. The present isolate under study produced significantly high amount of siderophore (78.7 ± 0.004 μmol). Besides enabling bacteria to grow in an iron-starved environment, siderophore confers an added advantage of increasing resistance to high arsenic concentration as compared to the non-siderophore producers [18]. Screening of comparative resistance efficiency of TA6 (78.7 ± 0.004 μmol) with a control strain *Acinetobacter guillourie* S02Ar2 (10.8 ± 0.003 μmol) and a mutant strain *Pseudomonas putida* (Lp10L02M) showed a significant difference. The isolate TA6 was able to resist higher concentration of arsenate in comparison to the mutant and control strain. Siderophore assisted increased resistance to arsenical compounds has been reported earlier [18]. The arsenic reducing efficiency of bacteria is also significantly influenced by varied siderophore concentration. High siderophore concentration confers higher resistance to arsenate as reported earlier [18].

**Fig. 7:**
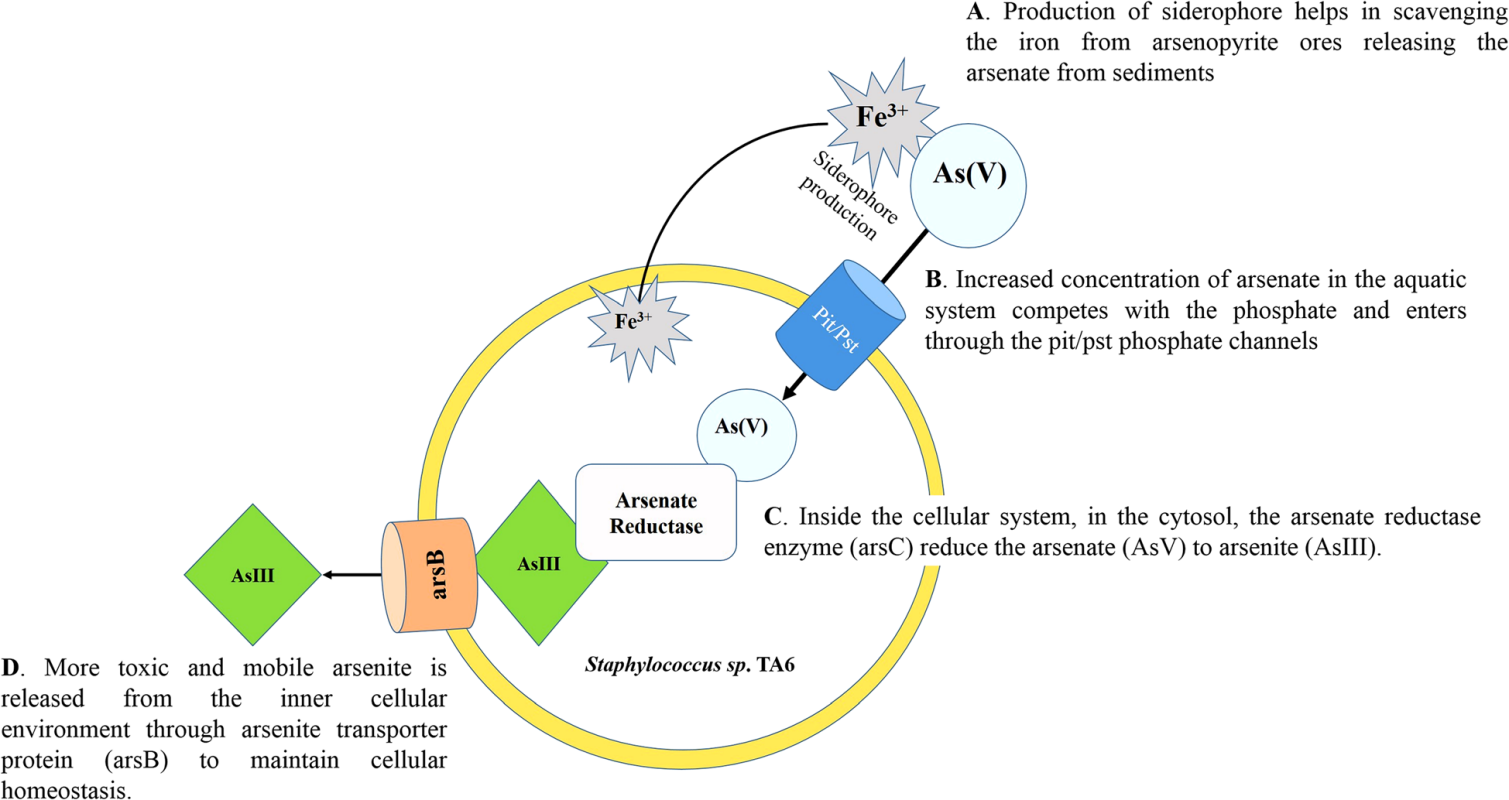
Graphical representation of tentative schematic mechanism of arsenic mobilization by *Staphylococcus sp.* TA6. The process can be catagorised in four sequential steps (A) production of siderophore to scavenge iron from arsenopyrite ores (B) Entry of arsenate through pit/pst phosphate transport channel into the cell (C) conversion of arsenate to arsenite and (D) efflux of arsenite to the surrounding environment.

Biotransformation assay revealed the present isolate as efficient arsenate reducer which actively catalyzed the reduction of As(V) to As(III) using an enzyme arsenate reductase encoded by *ars*C gene of the ars operon. Aerobic arsenate reduction is the most distributed detoxification mechanism present in the bacteria and the ars operon has been detected in more than 50 organisms within the domains of bacteria, yeast, and protist. The first recognized arsenate reductase gene was identified in a gram-positive *Staphylococcus* plasmid [38]. Since then there have been several reports of this gene in different bacterial species viz. *Staphylococcus sp., Thermus thermophiles* [48] *Bacillus sp., Shewanella sp.,* [38]. Analysis of NADPH coupled assay revealed the enzyme to be slightly acidic in nature with optimal activity at pH 5.5 and temperature of 50 °*C. michaelis* Menten kinetic constant, km was found to be 0.44 mM arsenate and V_max_ of 6395 umol/min/ml. A similar kinetics of this enzyme was reported from *Chrysiogenes arsenatis* with a Km value of 0.3 mM arsenate and Vmax of 7013 umol/min/ml [49]. The isolate displayed high reduction efficiency (88.2%) reducing the initial 2 mM arsenate [As(V)] added to arsenite [As(III)] over a period of 72 h. High activity of the enzyme leads to the conversion of arsenate to more mobile arsenite in the shallow aquifers that leads to its accumulation over a time period and could be one of the major reason for the increasing carcinogenic development in the northeastern region. Siderophore produced by the bacteria displaces iron from the iron-arseno compounds (arseno-pyrite) to releases the arsenic and thus aids in the mobilization of the sedimentary arsenate. Increased concentration of arsenate in surrounding milieu competes with the phosphate ion. As the structural homology of the arsenate is similar to phosphate it can enter the cellular system through *pit/pst* phosphate transporter channel [50]. Cellular arsenate is then converted to arsenite by arsenate reductase enzyme and soon effluxes out of the system through arsenite transporter channel to maintain the cellular homeostasis (Fig. 7) [30]. The increases in the concentration of both arsenite and arsenate in the aquatic system leading to eventually increased arsenic contamination in the Brahmaputra valley.

## Conclusion

Arsenic contaminated groundwater not only affects the human health but also crop health and food supply system when such water is used for irrigation. This leads to the accumulation of As in crops like rice grown extensively in the region and enhances the level of As in the soils rendering them unsuitable for agriculture. Our findings of the role of *Staphylococcus sp.* TA6 in the mobilization of As sheds further insight into the involvement of bacteria in arsenic distribution in the aquifer systems of the Brahmaputra valley. Further studies can provide information on other potential routes leading to increasing in As concentration in the environment and design effective strategies to make potable water safe.

## Additional files

Additional file 1: **Table S1.** Arsenic concentration of different districts of Northeastern States as reported by Singh (2004).Table S2 Groundwater profile from Jorhat District (As recorded during this study). (DOCX 19 kb)

Additional file 2: **Figure S1.** Map of the study area. (The map was prepared in Microsoft Office PowerPoint 2016). (TIF 3111 kb)

Additional file 3: **Figure S2.** Pure culture plate of *Staphylococcus sp.* TA6. (TIF 1857 kb)
