# Supplementary material for: Characterization of siderophore producing arsenic-resistant Staphylococcus sp. strain TA6 isolated from contaminated groundwater of Jorhat, Assam and its possible role in arsenic geocycle
Source: BMC Microbiol. 2018 Sep 4;18:104. doi: 10.1186/s12866-018-1240-6 (PMC6122220; doi:10.1186/s12866-018-1240-6)
Supplement: Supplementary file 1 — Table S1. Arsenic concentration of different districts of Northeastern States as reported by Singh (2004).Table S2 Groundwater profile from Jorhat District (As recorded during this study). (DOCX 19 kb) [file 12866_2018_1240_MOESM1_ESM.docx]

**Supplementary Tables**

**Table S1:** Arsenic concentration of different districts of Northeastern States as reported by Singh (2004) [1].

| **Sl. No.** | **State** | **Affected District** | **Arsenic Concentration (µg/l)** |
| --- | --- | --- | --- |
| 1 | Assam | Jorhat | 194 - 657 |
|  |  | Golaghat | 100-200 |
|  |  | Dhemaji | 100 - 200 |
|  |  | Lakhimpur | 50-550 |
|  |  | Karimganj | 50 - 300 |
|  |  | Nagaon | 112-481 |
|  |  | Dhubri | 100-200 |
|  |  | Darrang | 200 |
|  |  | Barpeta | 100-200 |
|  |  | Nalbari | 100-422 |
| 2 | Arunachal Pradesh | Papum Pare | 74 |
|  |  | Dibang Valley | 75 - 618 |
|  |  | West Kameng | 127 |
|  |  | East Kameng | 58 |
|  |  | Lower Subansiri | 63-159 |
|  |  | Tirap | 90 |
| 3 | Nagaland | Mokokchung, Mon, Wokha, Zunheboto | 50-278 |
| 4 | Tripura | West Tripura | 65 - 444 |
|  |  | North Tripura |  |
| 5 | Manipur | Thoubal | 798 - 986 |

1. Singh AK. Published in Proceedings of National seminar on Hydrology with focal theme on &quot; Water Quality &quot; held at National Institute of Arsenic Contamination in Groundwater of North Eastern India. Hydrology [Internet]. 2004 [cited 2017 Dec 8]; Available from: https://pdfs.semanticscholar.org/7e6a/5b6a2d3c05722a411dbc24d0526e7d30e4f4.pdf

**Table S2:** Groundwater profile from Jorhat District (As recorded during this study).

| **SL. NO.** | **NAME OF THE PLACE** | **DISTRICT** | **NO. OF SAMPLE COLLECTED** | **GPS** | **pH** | **ARSENIC CONCENTRATION (µg/L)** | **WELL DEPTH (ft.)** |
| --- | --- | --- | --- | --- | --- | --- | --- |
|  | Tanti Gaon (Titabor) | Jorhat | 6 | 27^0^57^//^N 94^0^16^//^E | 6.3-7.2 | 50-356 | 70-150 |
|  | Hatigarh | Jorhat | 3 | 27^0^78^//^N 94^0^27^//^E | 5.7-7.3 | 10-83 | 70-150 |
|  | Ladoigarh | Jorhat | 5 | 27^0^78^//^N 94^0^32^//^E | 6.2-7.1 | 30-170 | 70-150 |
|  | Meleng | Jorhat | 3 | 26^0^79^//^N 94^0^30^//^E | 5.8-7.2 | 10-60 | 70-150 |
|  | Bebejia Gaon | Jorhat | 4 | 26^0^58^//^N 94^0^18^//^E | 6.1-6.8 | 10-30 | 70-150 |
|  | Dakhinpat Gaon | Jorhat | 5 | 26^0^16^//^N 94^0^19^//^E | 6.7-7.3 | 50-112 | 70-150 |
|  | Gayan Gaon | Jorhat | 3 | 26^0^73^//^N 94^0^19^//^E | 6.3-7.5 | 10-30 | 70-150 |
|  | Chinamara | Jorhat | 4 | 26^0^70^//^N 94^0^23^//^E | 5.5-7.3 | 35-80 | 70-150 |
|  | Teok | Jorhat | 3 | 26^0^83^//^N 94^0^43^//^E | 5.0-7.8 | 90-129 | 70-150 |
