# Supplementary figures and images for: Characterization of siderophore producing arsenic-resistant Staphylococcus sp. strain TA6 isolated from contaminated groundwater of Jorhat, Assam and its possible role in arsenic geocycle
Source: BMC Microbiol. 2018 Sep 4;18:104. doi: 10.1186/s12866-018-1240-6 (PMC6122220; doi:10.1186/s12866-018-1240-6)

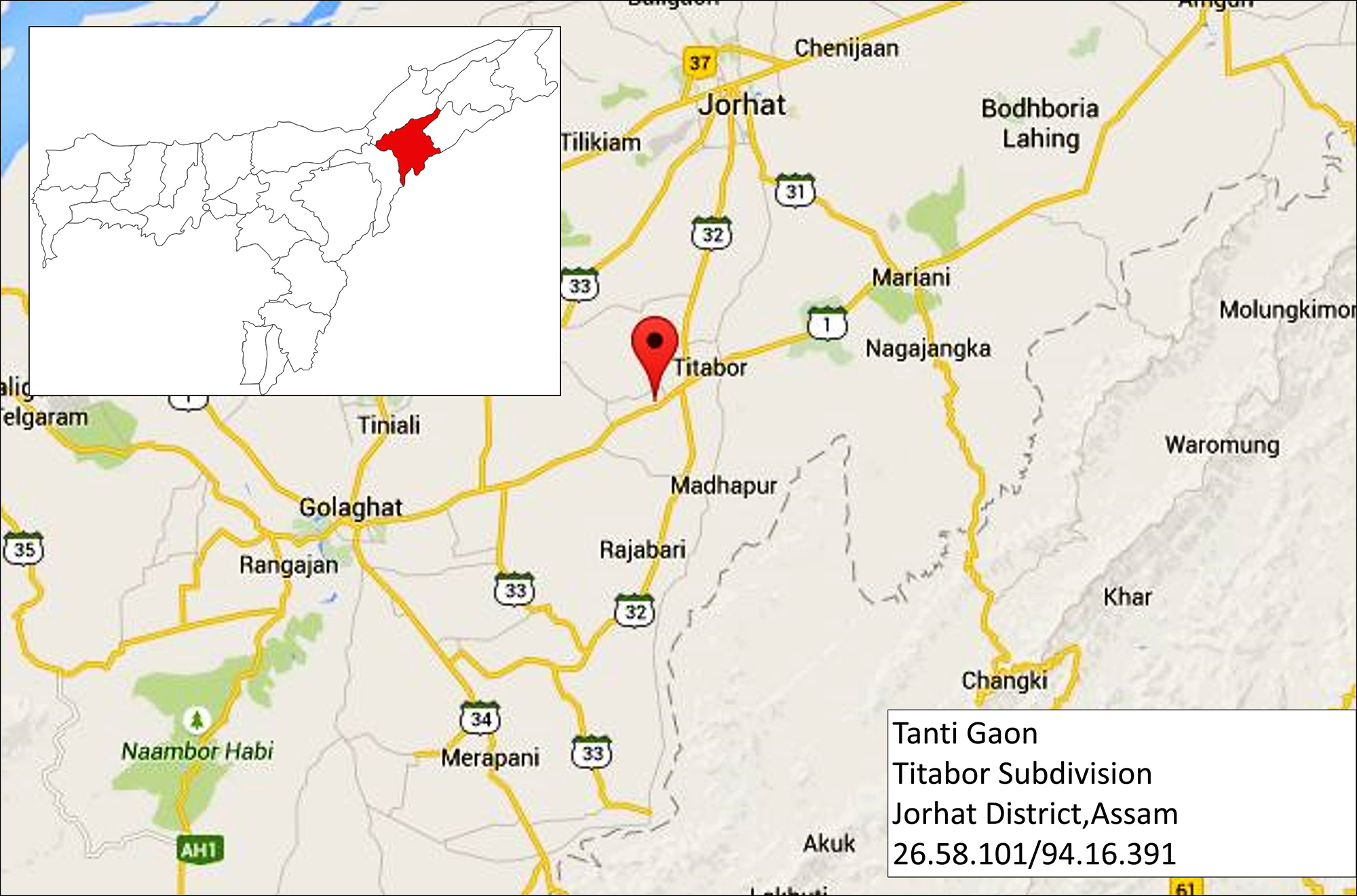

Supplement: Supplementary file 2 — Figure S1. Map of the study area. (The map was prepared in Microsoft Office PowerPoint 2016). (TIF 3111 kb) [file 12866_2018_1240_MOESM2_ESM.tif]

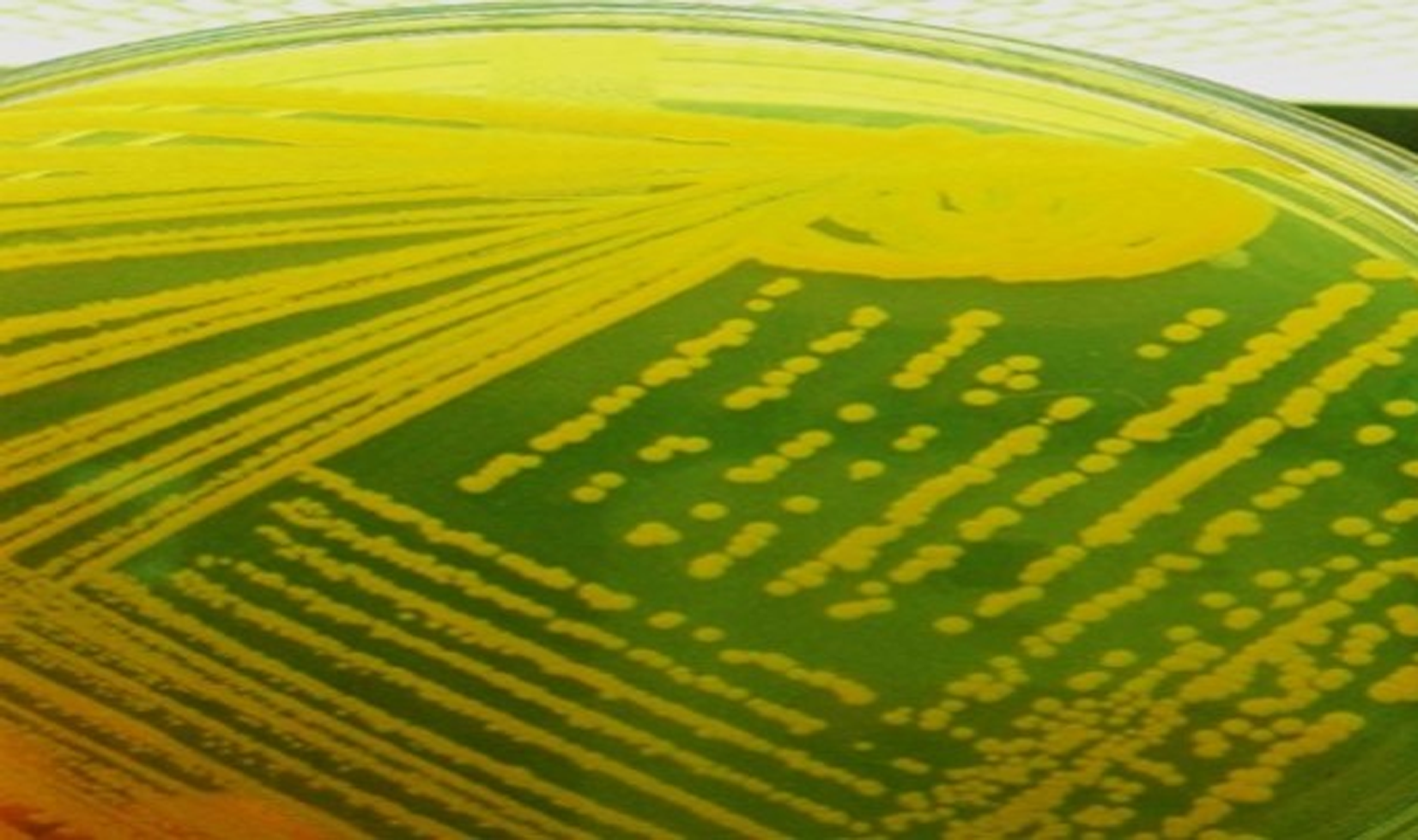

Supplement: Supplementary file 3 — Figure S2. Pure culture plate of Staphylococcus sp. TA6. (TIF 1857 kb) [file 12866_2018_1240_MOESM3_ESM.tif]
